# Removal of 2,4 di-nitrophenol by using modified spinel aluminate/chitosan nanoparticles composites

**DOI:** 10.1038/s41598-025-28057-8

**Published:** 2025-12-03

**Authors:** Amany N. Georgy, Mohamed A. Omar, Maysa R. Mostafa, Gehad G. Mohamed, Omar A. Fouad

**Affiliations:** 1 Department of Chemistry, Faculty of Science, Cairo University, Giza, 12613 Egypt; 2 Nanoscience Department, Basic and Applied Science Institute, Egypt-Japan University of Science and Technology, New Borg El Arab, Alexandria, 21934 Egypt

**Keywords:** Adsorption, 2,4-dinitrophenol, Magnesium aluminate–chitosan NPS, Isotherms–kinetics, Chemistry, Environmental sciences, Materials science, Nanoscience and technology

## Abstract

Recycling waste material into new products considered as a critical issue as it is cost-effective, and eco-friendly process. The basic material employed here for the synthesis of chitosan nanoparticles (CS-NPs) from the waste shrimp shells. Magnesium aluminates spinel (MAS) transparent ceramics doped with Ni^2+^ and Tb^3+^ ions synthesized through Sol-gel auto-combustion process is added. Mixing MAS and CS-NPs in varying ratios MAS/CS-NPs (1:9, 3:7, and 5:5), and then characterizing the resulting powder using X-ray diffraction and Transmission electron microscopy and BET surface area. The analysis reveals that the synthesized materials have high crystallinity with particle sizes ranging from 10 to 50 nm and mesoporous character with large surface area. Batch adsorption experimental technique was used to assess the adsorption capacity of the MAS/CS-NPs nanoparticles to adsorb 2,4- Dinitrophenol (2,4-DNP). At room temperature, variables such as contact time, pH, agitation rate, and MAS/CS-NP dose were taken into account. A clearance rate of about 91.3% was shown by the data at pH = 4, Nps dose = 0.03 g/L, and 200 rpm for 45 min. Moreover, it was established that the material could be used for three cycles with an 85% clearance rate. Analysis of the isotherms and kinetics revealed that the adsorption process entails a physical interaction consistent with the Temkin isotherm and conforms to pseudo-second-order kinetics, with diffusion boundary layer the rate-limiting phase.

**Supplementary Information:**

The online version contains supplementary material available at 10.1038/s41598-025-28057-8.

## Introduction

One of the gravest global concerns of the twenty-first century is the availability of clean and safe water. But because the population has grown so quickly and agriculture and industry have expanded, water pollution is an urgent environmental problem that that is increasingly receiving attention. It is an essential natural resource on the planet, used for agricultural, residential, industrial, and recreational purposes^1^. Water shortages and deterioration of water quality are currently global challenges that are worsening due to residential and agricultural activities, rapid industrialization, and increased energy demand^2^. Several organic, inorganic, and biological impurities (such as medicines, oils, phenols, pesticides, detergents, fertilisers, greases, microbiological pathogens, heavy metals, microplastics, and so on) have been identified as major water pollutants. Drinking water quality is deteriorating worldwide, particularly in underdeveloped countries. As a result, the scientific community must consider large-scale water filtration globally^3^.

Particularly, some industries discharge effluents containing a variety of pollutants like metals, dyes, phenols, phosphates, nitrates, herbicides, and medicines. Consequently, the aquatic ecosystem has been found to contain thousands of organic pollutants (such as organic dyes, phenols, biphenyls, pesticides, fertilizers, hydrocarbons, detergents, and pharmaceuticals), inorganic pollutants (such as heavy metals, phosphate, and nitrate), and biological pollutants (such as viruses, fungi, bacteria, amoebas, and algae)^4–7^. Adsorption, photocatalysis, ion exchange, membrane separation, and biodegradation are some of the varied techniques used to remove various categories of water pollutants. Finding environmentally friendly water treatment methods that are readily available, biocompatible, and non-toxic, with minimal or no harmful effects on the environment for generations to come, remains crucial^8^.

The adsorption method is commonly used in wastewater treatment to remove contaminants from water because it is simple, effective, inexpensive, and socially acceptable. An adsorption process consists of one or more adsorbates that are attached to an adsorbent via physical or chemical connections^9,10^.

Nitrophenols, among other phenolic chemicals, are hazardous to living organisms and increase oxygen demand in receiving waters. Most nitrophenols are resistant to biological treatment processes due to the presence of nitro groups in their structure, which provides them with high chemical stability and resistance to microbial destruction. Nitrophenols and similar compounds are released into the environment by pesticides and automotive exhaust gases, or as a consequence of the photochemical interaction of benzene with nitrogen monoxide in severely polluted air, and so appear as pollutants in water, wastewater, and the atmosphere^11^.

The most toxic substance in the phenolic family is 2,4-dinitrophenol (2,4-DNP), which has an LD 50 of 30 mg/kg body weight in rats^12^ and is regulated as a priority pollutant by the Clean Water Act^13^, implying the development of efficient and low-cost strategies for the removal of 2,4-DNP from aqueous solution.

Several strategies have been proposed for removing phenol and its nitro derivatives from water and wastewater, including chemical oxidation^14,15^, precipitation^16^, solvent extraction^17^, and adsorption^18,19^. The biological and chemical treatment of water and wastewater contaminated with nitrophenols, particularly 2,4-DNP, is a complex procedure. Many aerobic and anaerobic tests have been undertaken to determine the feasibility of biodegrading 2,4-DNP in the aqueous phase^20–23^.

Large colonies of bacterial strains have been seen to use 2,4-DNP as a carbon and nitrogen source^24,25^. Aside from that, the highly complex process of biodegradation presents significant challenges in optimisation and selection of favourable settings. In the long term, the procedure appeared to be extremely difficult to perform, and it may also produce surplus sludge that must be disposed. The adsorption procedure has been demonstrated to be efficient for the removal of phenolic compounds and their derivatives from water and wastewaters utilising a multitude of adsorbents such Lakhra coal^26^, zeolite^27^, sludge^28^, chitin^29^, fly ash^30^, and waste tires^31^.

Among biosorbents, chitosan, a derivative generated by N-deacetylation of chitin, is a naturally abundant and flexible biopolymer that plays an essential role in wastewater treatment. Chitosan is a polysaccharide made up of two types of monomeric units: one with an amino group (2-amino-2-deoxy-β-D-glucopyranose) and one with an acetamido group (2-acetamido-2-deoxy-β-D-glucopyranose). Additionally, it has a greater number of primary amines (-NH2) and hydroxyl groups (-OH), which provide active sites for effective adsorption. Chitosan’s peculiar structure allows for the simple removal of anionic and cationic contaminants such as reactive dyes, direct dyes, acids, metals, and so on^32–34^.

There are two types of nanoparticles: inorganic and organic. Because of the resistance of inorganic nanoparticles against harsh processing conditions, inorganic nanoparticles have become exceedingly important. The physical and optical stability along with the controllable optical properties of metal oxide nanoparticles, including magnesium oxide, zinc oxide, titanium oxide, and silver oxide, make them extremely desirable among inorganic materials. MgAl_2_O_4_ (MAS) spinel powder is an effective material with a variety of valuable properties, including outstanding refractoriness, good chemical inertness, mechanical strength, and high melting point (2135 °C). All these are due to the unique electronic, metallic, and structural characteristics of the material. These characteristics have led to the widespread use of MgAl_2_O_4_ spinel in numerous uses, including a humidity sensor and refractory material^35^. We can obtain spinel magnesium aluminate powders with improved properties, including high purity, chemical uniformity, and control of stoichiometry, fine particle size, narrow particle size distribution, and lowest collection of high sinter-activity particles, using a low-cost and simple preparation procedure^36^. Sol-gel, co-precipitation, hydrothermal, microwave-assisted combustion processing, micro emulsion, metal-organic processing, spray drying, freeze drying, and mechanochemical synthesis routes are only a few amongst those that have been suggested to synthesize MgAl_2_O_4_ spinel^37^.

Terbium ions are the most widely used rare earth (RE) dopant for materials. Trivalent terbium ions are applied in optical devices owing to their distinct optical characteristics, which occur in amorphous or polycrystalline hosts. Because of their intense luminescence at 550 nm, enabling direct contacts with silicon detectors, the devices are frequently used in radiography and other technological applications as thermal neutron detectors. Publications dedicated to spectral and kinetic studies of the terbium ions in various radiating systems and to the energy transfer mechanism show the persistent demand for these materials. The laws governing such relations have been developed, allowing for the design of various light detection techniques for RE ions in the presence of Tb^3+^ ions^38^.

So, the synthesis of green material based on chitosan as bioregion material waste as an adsorbent for the removal of organic material, such as 2, 4-dinitrophenol, is the main objective. In addition to ability to removal with high percentage in short time with low dosage amount without need any heating, with different ratios of MAS/CS-NPs composite (1:9, 3:7, 5:5). Using many techniques for determine the morphology and the surface analysis of the composite as by X-ray diffraction (XRD), FTIR, scanning and Transmission electron microscopy analysis and BET surface area. Study the factors that effect on the performance of this composite as an adsorbent in the removal of organic compound as 2,4 DNP from wastewater. Conditions affecting this process were optimized and studied along with describing the kinetic and isothermal models that fit the adsorption process.

## Experimental

### Materials

Magnesium nitrate (Mg(NO_3_)_2_.6H_2_O; Merck), aluminium chloride (AlCl_3_.6H_2_O; 97%; Sigma–Aldrich Chemical Co.), citric acid monohydrate (C_5_H_8_O_7_.H_2_O;98% ; Sigma–Aldrich Chemical Co). Hydrochloric acid (HCl; 37%), ethylene glycol (99%), ammonia solution (33%), nickel nitrate (Ni(NO_3_)_2_.6H_2_O), terbium oxide (Tb_4_O_7_;99%,), Sodium hydroxide (NaOH;99%), and 2,4-DNP(2,4-DNP dye C_5_H_4_N_2_O_5_; 184 g mol^− 1^) were purchased from Sigma–Aldrich Chemical Co., shrimp shells (From Egyptian fish shops(.

### Synthesis MAS/CS-NPs composite

#### Synthesis of MAS

In brief, stoichiometric amounts of materials doped with Tb^3+^ and Ni^2+^ ions were prepared (Mg_(1−χ)_Al_2_O_4_:0.0011Tb^3+^:χNi^2+^: χ = 0.00, 0.05, 0.1 g). The magnesium nitrate, aluminium chloride, and citric acid monohydrate stock solution for spinel synthesis was made by swishing these ingredients around in distilled water. Next, add the rare earth (dissolved terbium oxide in HCl, 37%) and nickel nitrate (Ni(NO_3_)_2_.6H_2_O, purity 99%). Add the appropriate amount of fuel (ethylene glycol) after that. The aqueous suspended solutions were made by adding NH_4_OH drop by drop; to achieve an appropriate homogeneity, this process needed to be continuously stirred for 40 min at 300 rpm. The solution’s pH was maintained at 11 during the precipitation process. After filtration, a water wash, and an overnight drying period in an oven at 120 °C, the co-precipitates were extracted. The dry precursor was calcined for eight hours at 1100 °C in a muffle furnace to produce MA spinel.

#### Synthesis of chitosan nanoparticles (CS-NPs) and MAS/CS-NPs composite

The shrimp shells were obtained from Egyptian fish shops, where a maximum of 2 kg of shrimp shell waste was washed using water until dirt that had stuck was removed. Then it was dried, powdered, and sieved by 50-mesh or 297 microns sieved, for further specification of sample preparation of CS from shrimp, then extraction of CS from shrimp shells waste was carried out in three steps, namely demineralization, deproteination, and deacetylation. The demineralization step was carried out with 1 M HCl at 90 °C for 3 h. It was then filtered and washed with distilled water to neutral pH. Qualitative tests were performed with AgNO_3_ until no white precipitate was left, i.e., Clions present had been gone. Dryness of the residue was at 60 °C. Deproteination was carried out with NaOH 4% at 65 °C for 2 h, and then washed with distilled water until pH was neutral. Qualitative testing was carried out by adding acetic acid until there was no precipitate formed. Then the residue was dried under the 60 °C temperature. Deacetylation was done using NaOH 50% through reflux at 90 °C for 8 h then filtering, washed with distilled water to pH neutral. Then the residue was dried further under a 60 °C until dry. Synthesis of CS-NPs was carried out by the ionic gelation method; CS powder was dissolved in 100 mL of 1% acetic acid to form CS solution, and was stirred for 1 h at room temperature. The solution was continuously stirred at room temperature for 2 h, and then dried using a spray dryer. The CS-NPs so formed was further characterized by xrd^39,40^, which was subsequently mixed in varying ratios with MAS spinel nanoparticles in ratios 1:9, 3:7, and 5:5 for MAS spinel to chitosan nanoparticles, respectively.

### Characterization

Patterns from X-ray diffraction (XRD) are used to characterise the sample. The Scherrer equation (Eq. 1) was used to calculate the crystallite size of the spinel powder

1 $$\:{\beta\:}\left(2{\uptheta\:}\right)=\frac{{\kappa\:}{\lambda\:}}{{L}\mathbf{cos}\:{\theta\:}\:0}$$

Where λ is the wavelength (= 0.15406 nm), θ_o_ the Bragg angle, Ƙ a constant (= 0.9), and Լ is the crystallite size. The halfwidth of the diffraction line ($\:\beta\:\left(2{\uptheta\:}\right)$ (in radians) was taken as the experimental half-width ($\:\beta\:$_exp_) and was corrected for experimental broading ($\:\beta\:$_instr_) according to (Eq. 2)

2 $$\:\:{\beta\:}\left(2{\uptheta\:}\right)=({{\beta\:}}_{{e}{x}{p}}^{2}-{{\beta\:}}_{{i}{n}{s}{t}{r}}^{2}{)}^{1/2}$$

($\:\beta\:$_instr_) was measured experimentally by a silicon sample. Four diffraction peaks (3 1 1), (4 0 0), (1 1 1), and (2 2 0) were chosen for measuring the size of crystallites and the half-widths were calculated by the software installed on the diffractometer. Acetone was used to disperse the granules in order to create TEM samples. A drop of the suspension was placed on holey carbon film supported on copper grids using a high resolution transmission electron microscope (HR TEM; JEOL 2010 F, Microanalysis Centre Research Institute in Giza, Egypt) after ultrasonic oscillation was applied for one hour to reduce aggregation^41–45^.

### Dinitrophenol assays in a batch system

The adsorbent removed the dye using the batch method. Batch adsorption studies were performed using amount of adsorbent in dye solution at a constant temperature of (25 ± 1 °C). Once the required duration had elapsed, a spectrophotometric method was employed to determine the dye’s concentration under examination. Using spectrophotometry, the ultimate dye concentration was determined to match the dye’s maximum at 360 nm. For dyes and adsorbents, several variables were investigated, including pH level, contact time, beginning dye concentrations, amount of adsorbent and the effect of different ratios of the composite. The results of these studies were used to determine the best practices for extracting the greatest amount of dye from aqueous solutions. The following equation (Eq. 3) was used to calculate the dye clearance rate.

3 $$\:\%\:Removal=\frac{\left({C}_{0}{-C}_{e}\right)}{{C}_{0}}\times\:100$$

Where, *C*_*0*_ is the dye’s initial concentration (mg L^− 1^), *C*_*e*_ ( equilibrium concentration) is the dye concentration after adsorption (mg L^− 1^). (The following equation (Eq. 4) can be used to determine the adsorbent’s *q*_*e*_ max.adsorption capacity of the adsorbent) (mg dye per g dry adsorbent) adsorption capacity:

4 $$\:{\:q}_{max}=({C}_{0}-{C}_{e})\frac{V}{w}$$

Where, *V* (in liters) is the solution volume and *w* (in grams) the amount of dry adsorbent^9,44^.

## Results and discussion

### X-ray diffraction analysis

**Fig. 1:**
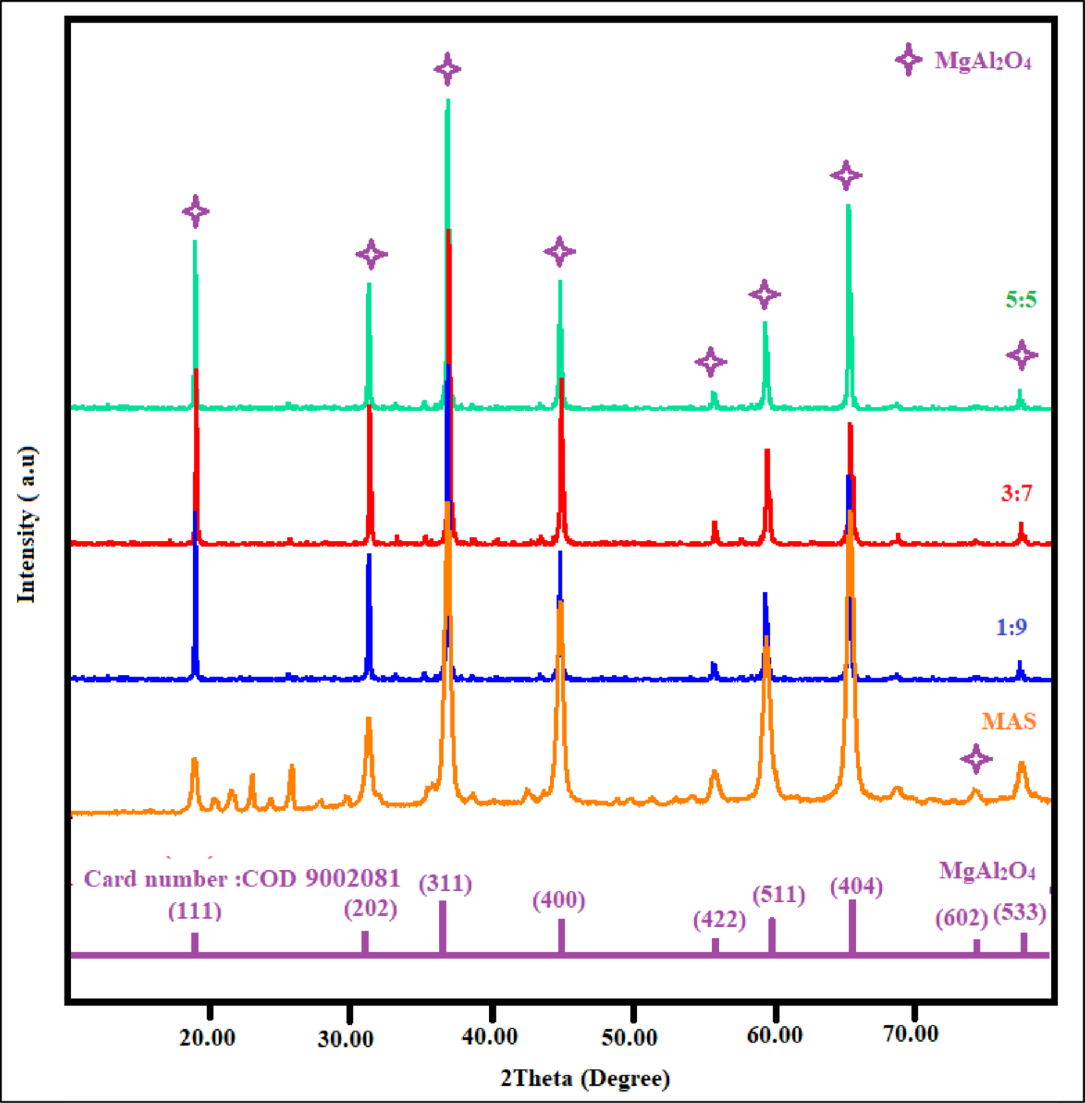
XRD patterns of MAS and MAS/CS-NPs.

The materials were scanned between 10° and 80° in the 2θ range. The MAS XRD pattern seen in Fig. 1 shows diffraction peaks at 2θ = 18.96°, 31.21°, 36.79°, 44.74°, 55.57°, 59.26°, 65.14°, 74.0°, and 77.22°, which correspond to (111), (202), (311), (400), (422), (511), (404), (602), and (533). As per the card number (COD: 9002081) and space group F d -3 m (227), these strong and sharp peaks validate the creation of the MAS spinel phase^45^. The XRD patterns of MAS/CS-NPs in different ratio (1:9, 3:7, 5:5) showed that the amorphous chitosan peak (around 2θ ≈ 10–20°) usually disappears in the X-ray diffraction (XRD) analysis this is because of the strong, sharp diffraction peaks of crystalline MAS. For instance, Mohammed K. Al-Hussainawy et al.^46^ reported that in their Fe_3_O₄–chitosan nanocomposite the chitosan peak at 10.55° vanished completely, with only magnetite peaks remaining visible. Similar to this, Dalia Amer Ali et al.^47^ found a broad peak in a range of 2θ = 20–22.53°; these peaks were completely absent in the composite, but because of the high crystallinity of magnetite, reflections related to FeO₄ predominate^12^. This is because of the strong interaction between MAS and chitosan, which lowers the chitosan matrix’s intermolecular hydrogen^46,48^.

### Transmission electron microscopy (TEM)

**Fig. 2:**
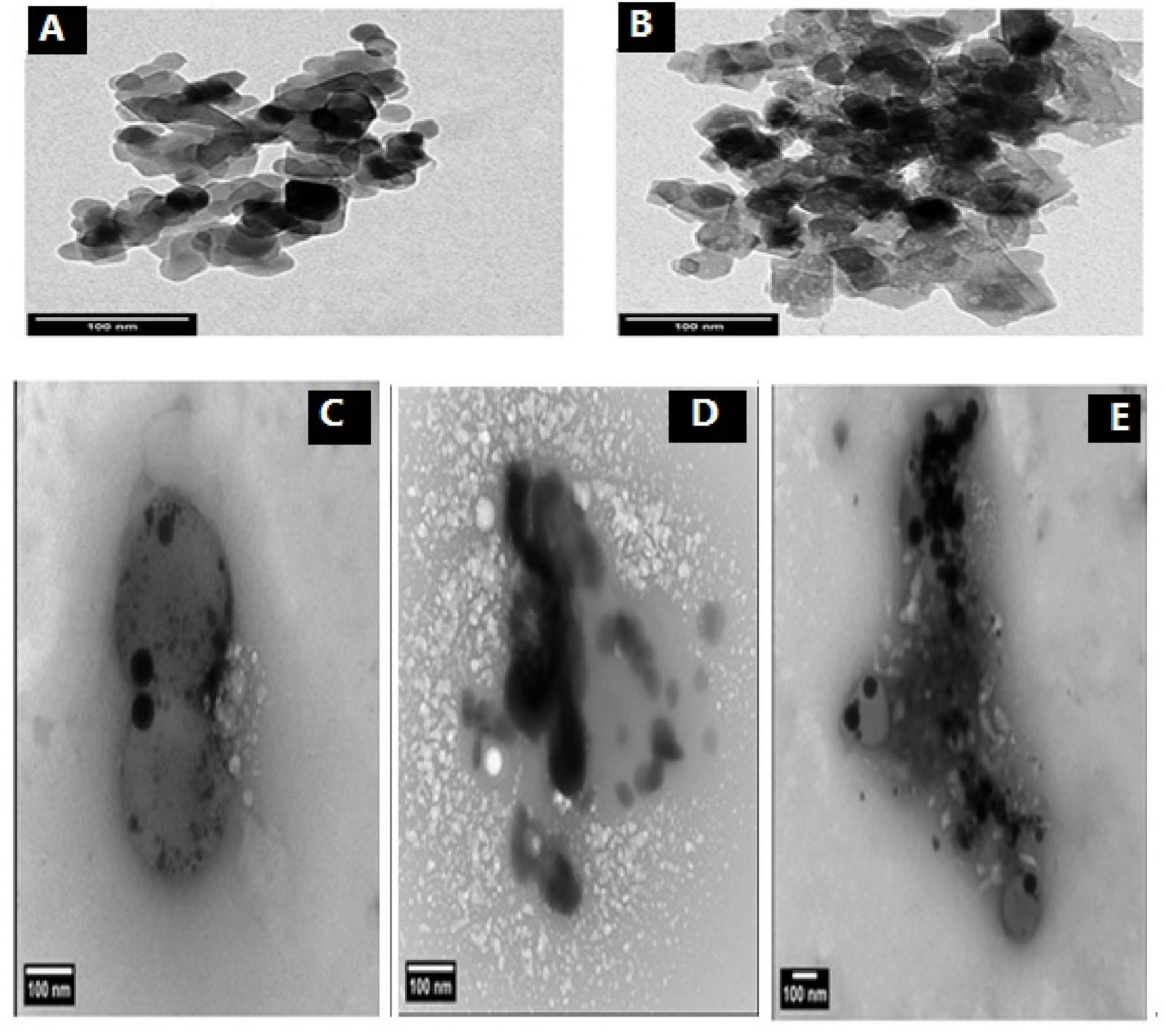
TEM micrographs of MAS and MAS with different ratio of CS-NPs (1:9, 3:7, 5:5). **A** and **B** for MAS and **C** for (5:5) of MAS -CS NPs, **D** for (3:7) MAS -CS NPs and **E** for (1:9) of MAS -CS NPs.

Transmission electron microscopy (TEM) is another useful technique for characterizing nanomaterials, providing quantitative insights into their size and shape. Figure 2A and B demonstrate that the MAS nanoparticles possess a well-defined cubic crystal structure. The average particle size was found to be between 22 and 35 nm. Also, the TEM micrographs of the MAS-CS- NPs produced by MAS with varying ratios of CS-NPs (1:9, 3:7, and 5:5) are shown in Fig. 2C–E. A TEM image indicates that the CS-MAS-NPs’ primary morphology is spherical particle of MAS dispersion into CS nano particle and was noted also that by increasing the ratio of the MAS into CS (1:9, 3:7, and 5:5) the ratio of the dark spherical increasing into the CS nano particle with a varying in the particles size in range of about 10–50 nm, this result confirm the nano scale of the synthesized materials and also the dispersion of MAS into CS^49,102^ as shown in Fig. 2C–E.

### FTIR analysis

**Fig. 3:**
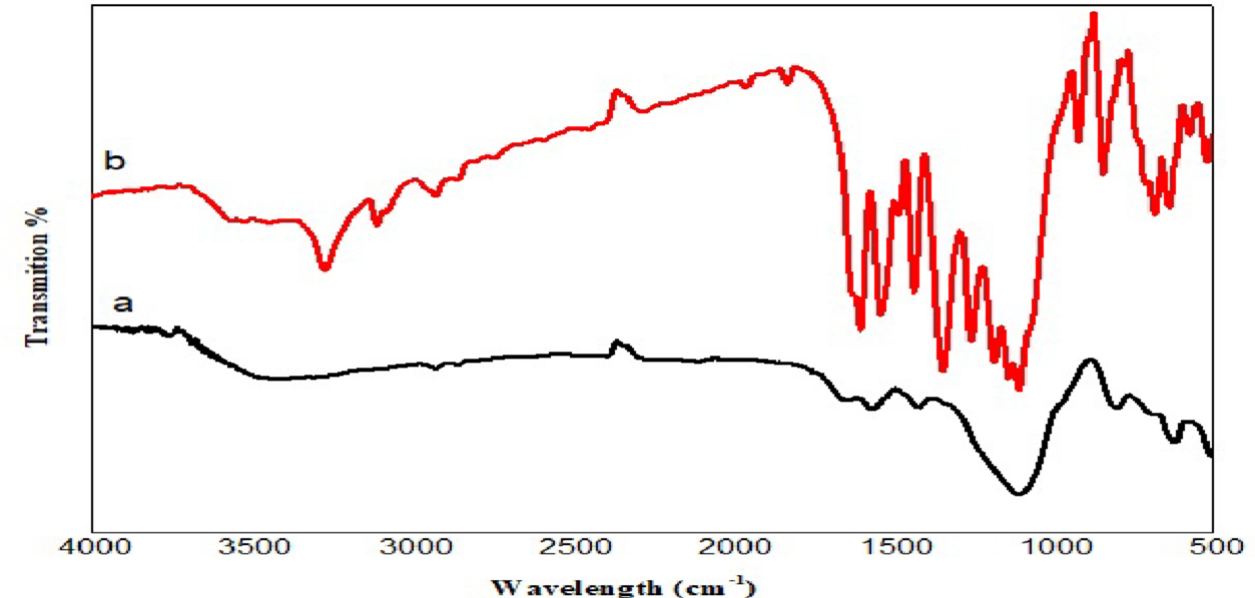
FTIR spectra of (**a**) MAS/CS-NPs before adsorption and (**b**) MAS/CS-NPs after adsorption.

Figure 3, shows FTIR spectra at two levels, prior and under the catalytic action. Figure 3A, Mg–O and Al–O stretching vibrations were identified as being responsible for the gained signal below 600 cm^− 1^. Signals that have been observed at 620 and 470 cm^− 1^ were thus associated with explained bonding^50^. Asymmetric C-O stretching vibrations were associated with signals that have been observed between 1000 and 1100 cm^− 1^. Signals in the 1300–1400 cm^− 1^ range were attributed to C–H bending vibration asymmetric vibration. Evidence for the presence of N–H bending vibrations (amide II band) of chitosan amino groups was achieved via vibration at 1557 cm^− 1^^51,52^. N–H stretching vibrations of the amino groups in the chitosan were responsible for the signal at 3398 cm^− 1^. Surface presence of the hydroxyl group, i.e., the -OH group, was evidenced through the vibration detected at 3762 cm^− 1^^53^.

### Surface morphology (SEM)

**Fig. 4:**
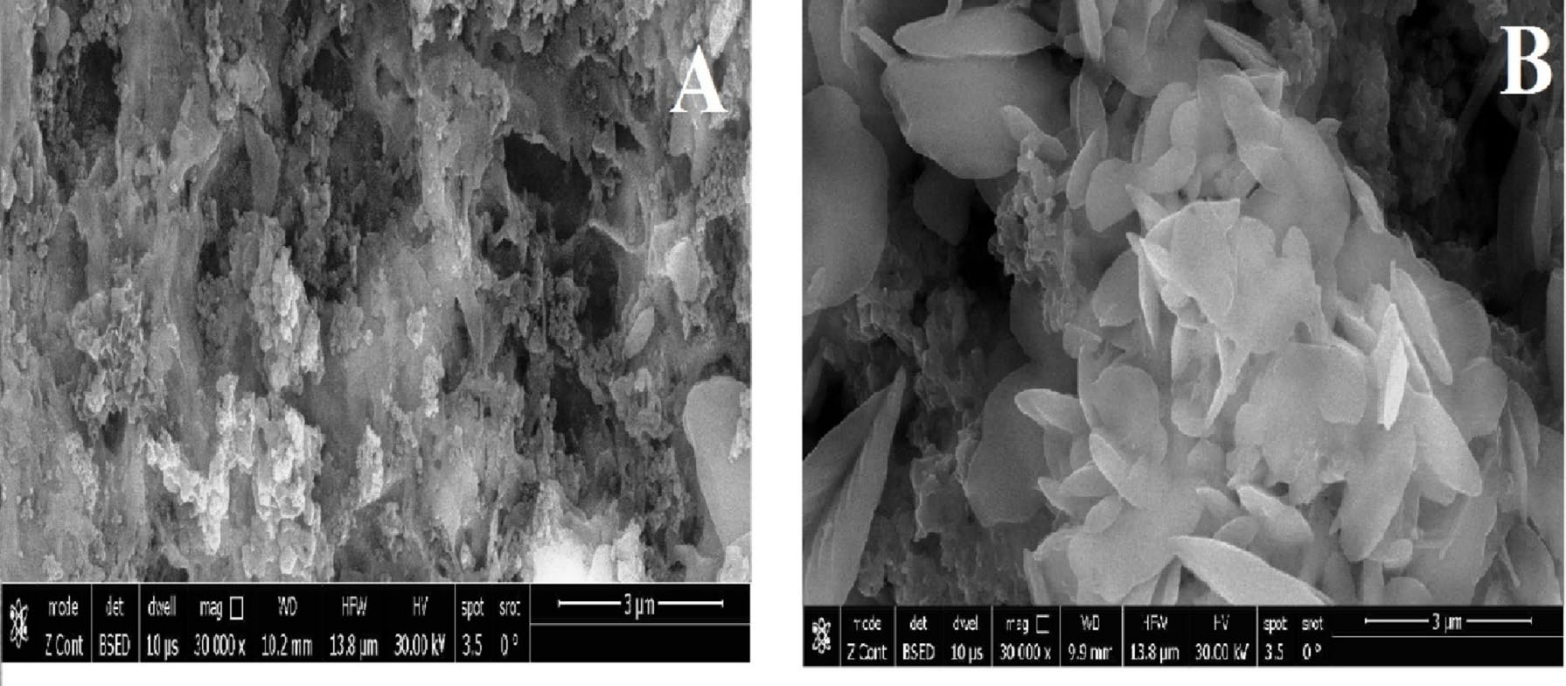
SEM images of (**A**) MAS/CS-NPs before adsorption and (**B**) MAS/CS-NPs after adsorption.

**Fig. 5:**
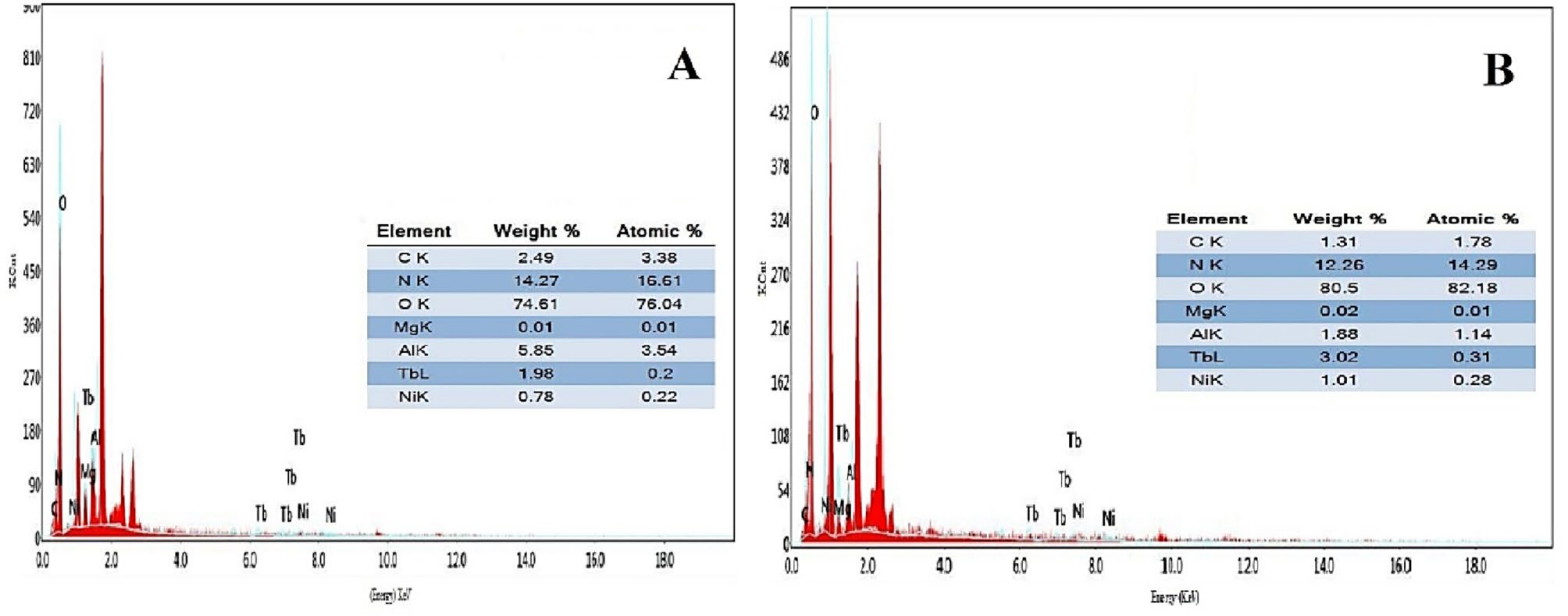
EDX graphs of (**A**) MAS/CS-NPs before adsorption and (**B**) MAS/CS-NPs after adsorption.

The dramatic morphological changes on the surface before and after adsorption are evident in the SEM micrographs. Figure 4A, The MAS/CS-NPs first exhibit a porous, rough surface with uniformly dispersed, generally spherical or irregular nanoparticles (~ 50–200 nm), revealing a large surface area that favors adsorption. Successful doping and synthesis of Tb and Ni in MgAlO₄ are indicated by EDX elemental analysis Fig. 5A, demonstrating the existence of necessary elements (Mg, Al, Tb, Ni, C, N, and O) with homogeneous distribution. Figure 4B Post-adsorption images demonstrate the development of a composite structure with increased surface roughness and porosity, as well as nanoparticles coverage with some aggregation. Following adsorption Fig. 5B, elemental spectra indicate the presence of Ni, Tb, Mg, Al, C, N, and O with extra signals for nitrogen and oxygen, confirming the grafting of 2,4-dinitrophenol molecules onto the surfaces of the nanoparticles. All considered, morphological and elemental changes indicate that the nanoparticle surfaces have been successfully modified and adsorbed^50,55,103^.

### Brunauer-Emmett-Teller (BET) analysis

In accordance with the definition of physisorption isotherms provided by IUPAC, the results of the nitrogen adsorption test demonstrated that the synthesized nano MAS/CS-NPs displayed a type IV-(a)-H3 hysteresis loop isotherm, which is characteristic of mesoporous materials. The pore size distribution, which is a more detailed analysis of the porosity than total pore volume, was determined from the adsorption data. According to the IUPAC classification, pores are defined as micropores (width < 2 nm), mesopores (width 2–50 nm), and macropores (width > 50 nm). The specific surface areas, total pore volume, and average pore radius of MAS and MAS with varied ratios of CS-NPs (1:9, 3:7, and 5:5) are provided in Table 1. The calculated average pore radii for all samples fall within the range of 3.2 to 4.6 nm, which unequivocally confirms the mesoporous nature of the adsorbents. This table shows that the surface area of the synthesized materials is well and significantly enhanced with the increase in the ratio of chitosan in the MAS/CS composite. The average pore radius also follows the same increasing trend, further reinforcing that all the synthesized materials are mesoporous^10,56^.

**Table 1:** BET analysis of MAS/chitosan adsorbent.

Sample	MAS	MAS/Chitosan ratio		
		5:5	3:7	1:9
BET surface area (m^2^g^− 1^)	60.79	31.649	42.009	82.881
Total pore volume (cm^3^g^− 1^)	0.081	0.051	0.077	0.096
Average pore radius (nm)	3.281	3.197	3.678	4.645

### Adsorption measurements using batch method

In order to identify the ideal parameters demonstrating the maximum removal effectiveness of the 2,4-DNP dye under investigation, researchers have examined factors that affect dye removal from aqueous solutions, including pH, adsorbent dosage, initial dye concentration, and contact time.

#### Effect of pH

**Fig. 6:**
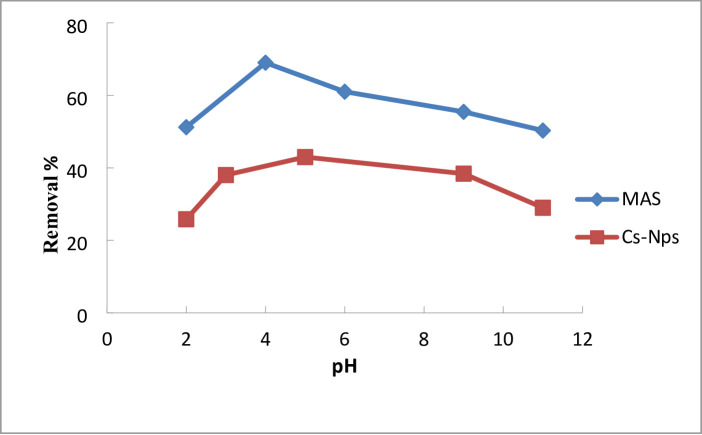
Effect of pH on the removal of 2,4-DNP dye.

Of the critical parameters governing adsorption efficacy, solution pH exerts a profound influence by modulating the surface charge of the adsorbent and the ionization state of the adsorbate. This relationship was quantified for the removal of the anionic 2,4-DNP dye using a MgAl_2_O_4_:0.0011Tb^3+^:(0.1)Ni^2+^ nanoadsorbent, where experiments were conducted with 3 ppm dye and 0.03 g adsorbent over a pH range of 2–11 for 120 min at 25 °C. As shown in Fig. 6 the maximum removal efficiency of MAS equal 73.83% that was observed at pH 4, attributable to the protonation of the adsorbent surface, which generates a positive charge favorable for electrostatic attraction to the negatively charged dye molecules. For chitosan, the adsorption behavior of chitosan powder is significantly influenced by the pH of the solution. In contrast to a 29% removal efficiency in alkaline settings (pH = 11), it achieves its highest removal efficiency of 42.8% in acidic conditions (pH = 5) and 42.02% at pH = 4. Conversely, increasing the pH introduced an excess of OH⁻ ions that competed for adsorption sites and increased the negative surface charge on the adsorbent, resulting in electrostatic repulsion of the dye and a subsequent decline in removal efficiency to below 50% in alkaline conditions. This demonstrates that pH-dependent electrostatic interactions between the adsorbent and the adsorbate primarily drive the adsorption process^57,58^.

#### Effect of contact time

**Fig. 7:**
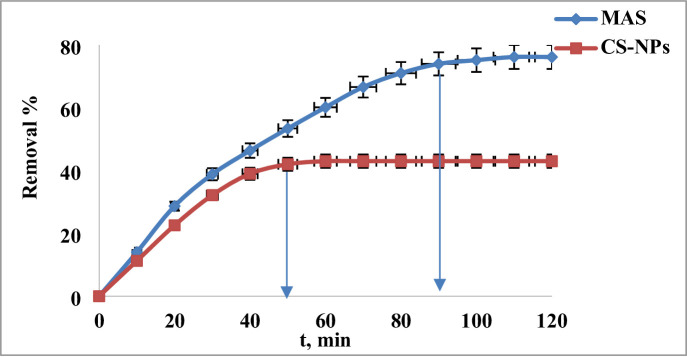
Effect of time of contact on the removal of ppm of 2,4-DNP dye at pH 4 using 0.03 g of MAS and CS-NPs nanopowder at different time.

As seen in Fig. 7, the effect of contact duration on 2,4-DNP adsorption was examined using a range of contact times (from 0 to 120 min), 3 ppm of dye solution, a pH of 4, a temperature of 25 °C, and an adsorbents dosage of MAS and CS-NPs of 0.03 g. The results showed that the elimination percentage of 2,4-DNP dyes adsorbed was 73.83% in case of MAS within 90 min and 42.02%, for CS-NPs within 50 min and that no further significant adsorption was observed after that. This is because the MAS and CS-NPs nanopowders’ open pores and unused adsorption sites gradually fill up and slow down over time until they achieve complete saturation after accurate minutes^9,59^.

**Fig. 8:**
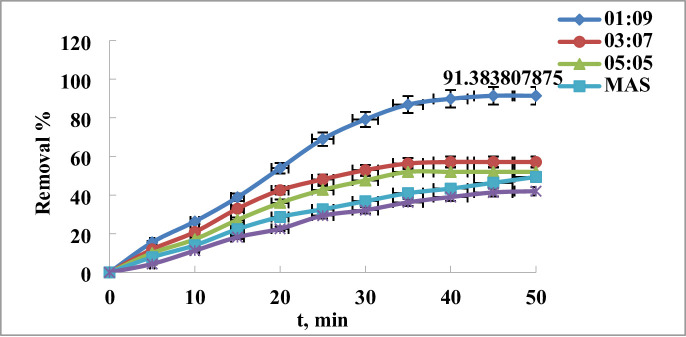
Effect of time of contact on the removal of 3 ppm of 2,4-DNP dye at pH 4 using 0.03 g of (MAS/CS-NPs (1:9, 3:7, 5:5), MAS, CS-NPs) at different time.

The technique was used to create a variety of MAS/chitosan nanopowder. For a material to be successful as an adsorbent, it must have a quick and quantifiable adsorption rate. An investigation was conducted to examine the influence of contact duration on the adsorption of dye, with time intervals ranging from 5 to 60 min. Figure 8 illustrates the effect of the modified nanoparticles MAS/CS-NPs in the effeceincy of the removal that the clearance percentage of 3 ppm dye concentration is almost 91.3% in case of ratio 1:9, 57.13% for ratio 3:7 and 51.99% for ratio 5:5 at 45 min using 0.03 g dose and pH of 4.

So, the optimum condition for the removal is done by 0.03 g dose and pH of 4 using ratio 1:9 MAS/CS-NPs the removal % will be 91.3% within 45 min.

#### Effect of adsorbent dosage

**Fig. 9:**
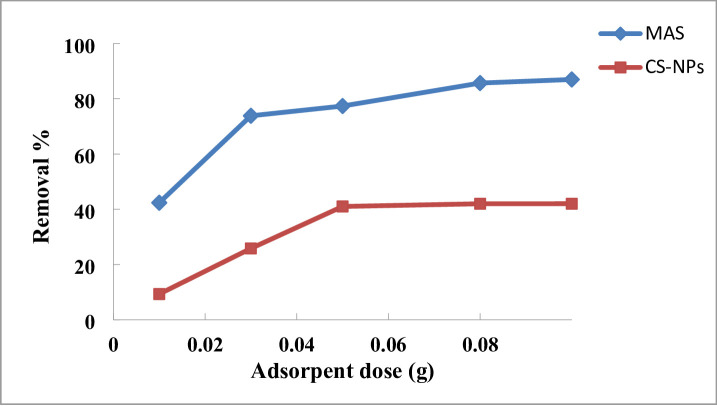
Effect of adsorbent dose of MAS and CS-NPs on removal of 3 ppm of 2,4-DNP dye.

As illustrated in Fig. 9, the amount of MAS and CS-NPs adsorbent nanopowder dosage that was used varied from 0.01 to 0.1 g at a dye concentration of 3 ppm at contact time 90 and 50 respectively. The removal effectiveness of 2,4-DNP dye increased from 42.36 to 86.98% when the dosage of doped MAS nanopowder adsorbent was raised from 0.01, 0.03, 0.05.0.08, to 0.1 g at pH 4, and for CS-NPs, the removal effectiveness of 2,4-DNP dye improved from 9.31 to 42.02%. The increase in sorption active sites at the adsorbent surface with increasing dosage explains this outcome. Despite the elimination efficiency rising to the maximum tested dosage of 0.1 g, a dosage of 0.03 g was chosen as the practical optimum for subsequent investigations. The conclusion was predicated on a cost-benefit study, indicating that 0.03 g achieved a notable removal efficiency (~ 73.83%) while employing the nanomaterial more than three times as effectively as 0.1 g. This method highlights the economic viability and sustainable use of the adsorbent without significantly sacrificing performance^58^. –^59^.

**Fig. 10:**
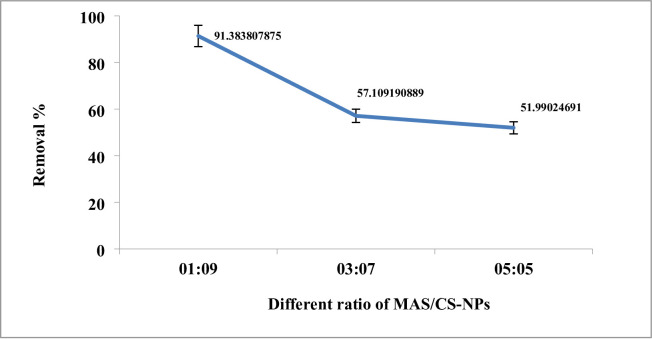
Effect of adsorbent dose of MAS with different ratio on the removal efficiency.

Using the mentioned technique, a number of MAS/chitosan nanopowders were made; Fig. 10, demonstrates the impact of varying MAS and CS-NP ratios (1:9, 3:7, and 5:5) on the materials’ ability to remove 2,4-DNP dye in 3 ppm at pH 4 over 45 min and with an adsorbent dose of 0.03 g. It is evident that the removal effectiveness of 2,4-DNP dye decreases as the weight fraction of chitosan decreases; in other words, adding chitosan improves the materials’ efficiency to remove the dye till reached removal efficacy 91.3% for MAS /CS-NP ratios (1:9) over 45 min at a steady temperature of 25 °C^60,61^.

#### Effect of the initial dye concentration

**Fig. 11:**
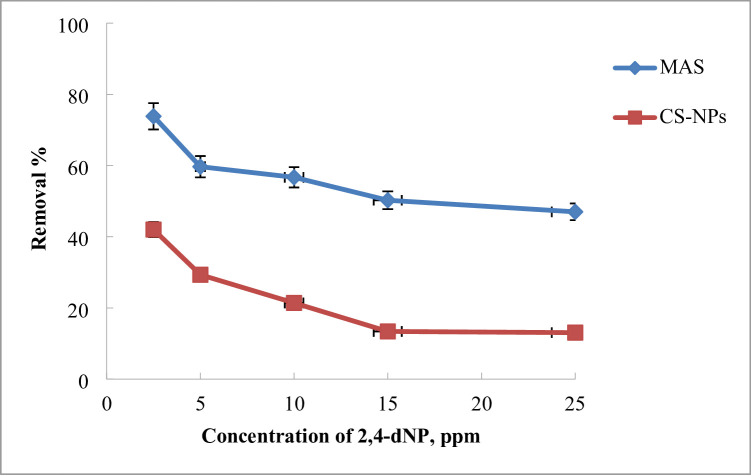
Effect of concentration of 2,4-DNP dye using MAS and CS-NPs nanopowder.

The phenomenon of dye adsorption, which includes the transfer of mass, occurs when dye molecules accumulate at the boundary between a solid and a liquid. The study focused on analyzing the impact of the initial dye concentration while holding the other parameters constant. As shown in Fig. 11, the removal percentage for MAS decreases from 73.83 to 47.9% and for CS-NPs decreases from 42.02 to 13.06% when the initial dye concentration increases from 2.5 to 25 ppm. The higher initial dye concentration led to a decrease in the removal percentage because there were fewer active sites on the adsorbent, which is particularly noticeable when the initial concentration of 2,4-DNP is greater than 5 ppm. The barrier to mass transfer between the liquid phases (the dye, water, and other interfering substances) and the solid phase (the MAS or CS-NPs adsorbent) is another factor that causes the removal percentage to decrease. However, the MAS or CS-NPs provided a critical impetus to overcome this obstacle in the mass transfer, which resulted in a decrease in the barrier to mass transfer and an improvement in the efficiency of dye removal as the initial concentration decreased. Similarly, previous studies have shown that as the concentration of 2, 4-DNP rises, the percentage of Dinitrophenol adsorbed decreases because there are insufficient surface sites to support the dye’s concentration^60,61^.

**Fig. 12:**
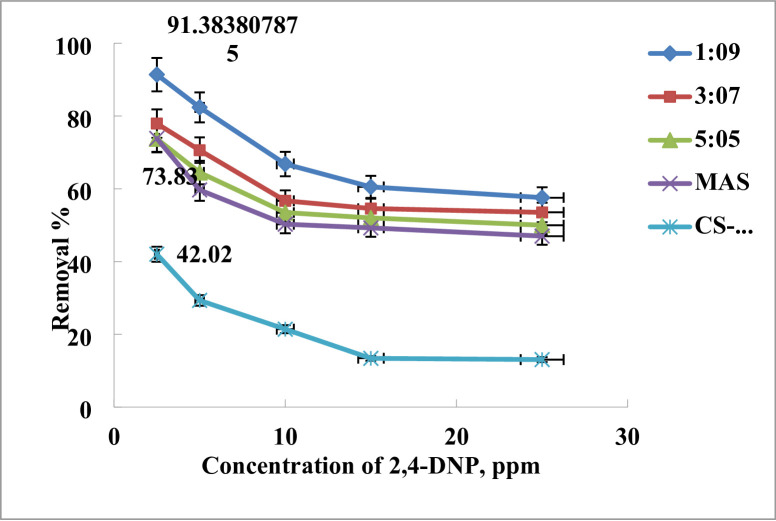
Effect of removal of 2,4-DNP dye using MAS, CS-NPs and different ratios of MAS/CS-NPs.

The aforesaid procedure was used to manufacture a variety of MAS/chitosan nanopowders; the initial dye concentration is crucial for determining the adsorption potentials of the adsorbents. Figure 12, used various ratios of MAS and CS-NPs (1:9, 3:7, and 5:5) nanopowders to show how changes in the concentration of 2,4-DNP dye affected the percentage of elimination. Evidently, the overall trend is that as the weight% of chitosan decreases, so does the 2,4-DNPdye’s removal efficacy. So according the pervious results for the pH effect, contact time, dose and initial concentration it was relive that the optimum material for removal of 2,4-DNP dye by high efficiency was MAS/CS nanocomposite by weigh ratio (1:9) so, the isotherm, kinetics and reusability studies was done into this nanomaterial. So, the ratio (1:9) MAS/CS nanocomposite have an ability to removal the dye up to 15 ppm with about 60% efficiency^62–65^.

### Adsorption kinetics

Adsorption kinetic describes the mechanism of adsorption processes which in turn controls the equilibrium time and mass transfer of the adsorbate. It was found that the non-linear form has an advantage over the linear model because the distribution of the error remains unchanged, and the kinetic factors are fixed on the same ordinates and axis. The optimal model was assessed using R^2^ correlation coefficients, and the χ ^2^ test, with the superior model exhibiting the highest R^2^, and the lowest χ ^2^ value.

The experimental data of 2,4-DNP adsorption was investigated using the pseudo-first order as Eq. (5), pseudo-second order as Eq. (6) and Intra particle diffusion models shown in Eq. (7)^21,66–70^.

5 $$\:\text{L}\text{i}\text{n}\text{e}\text{a}\text{r}\:{log}\left({{q}}_{{e}}-{{q}}_{{t}}\right)={log}\:{{q}}_{{e}}-\left(\frac{{{k}}_{1}}{2\cdot\:303}\right){t}\:,\:\text{n}\text{o}\text{n}\text{l}\text{i}\text{n}\text{e}\text{a}\text{r}\:{{q}}_{{t}}={{q}}_{{e}}{(1-{e}}^{{k}1{t}})$$

6 $$\:\:\text{L}\text{i}\text{n}\text{e}\text{a}\text{r}\:\frac{{t}}{{{q}}_{{t}}}=\left(\frac{1}{{{k}}_{2}{{q}}_{{e}}^{2}}\right)+\left(\frac{1}{{{q}}_{{e}}}\right){t}\:,\:\:\:\text{n}\text{o}\text{n}\text{l}\text{i}\text{n}\text{e}\text{a}\text{r}\:{{q}}_{\mathbf{t}}=\frac{{\:{k}}_{2}{{q}}_{{e}}^{2}\:{t}}{1+{{{t}\:{q}}_{{e}}{k}}_{2\:\:\:}}$$

7 $$\:{{q}}_{{t}}={{k}}_{{i}}{{t}}^{0.5}+{C},$$

where q_e_ and q_t_ are the amounts of adsorbed in equilibrium and at time t, respectively, k_1_, k_2_, and k_id_ are the equilibrium rate constant of pseudo-first order adsorption (1/min), pseudo-second order adsorption (g/mg.min), and intra-particle diffusion model (mg/g.min^1/2^ ), and C is the intercept.

**Fig. 13:**
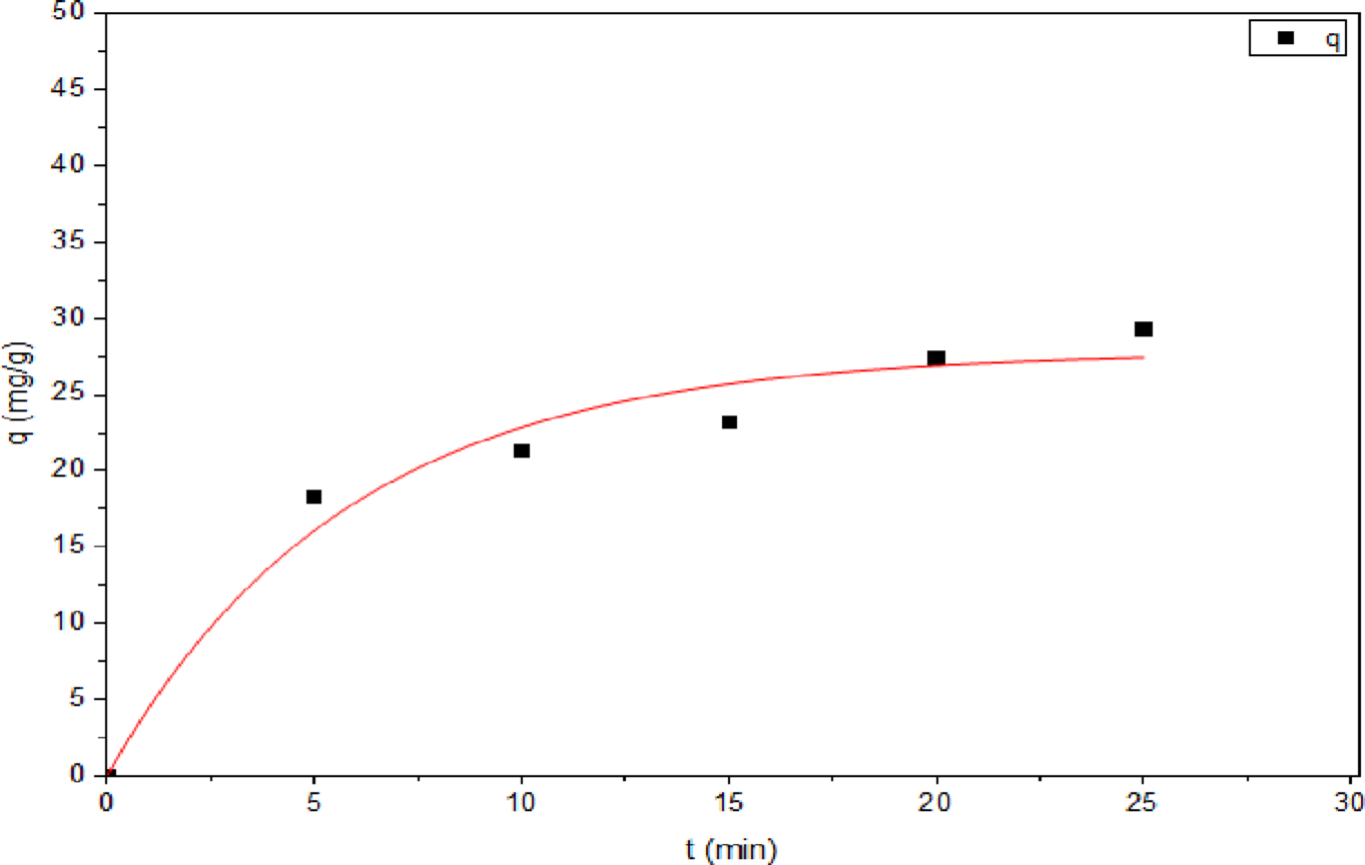
Pseudo- first order model for the adsorption.

**Fig. 14:**
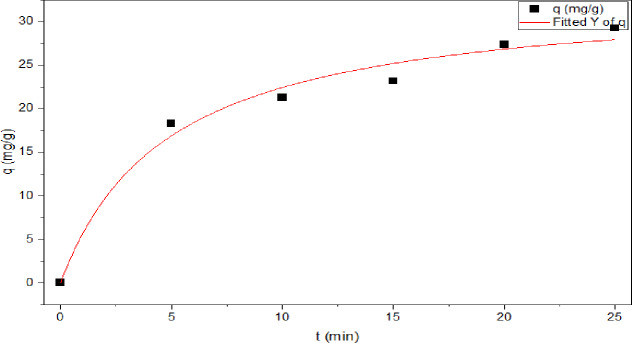
Pseudo- second order model for the adsorption.

For the Pseudo-First-order model, Fig. 13; Table 2 illustrate that plotting log (q_e_-q_t_) against time which reveals a linear relationship, with an R^2^ value of 0.960 and a rate constant k_1_ of 0.137 min^-1^. For the pseudo-second-order model, Fig. 14, plotting t/qt against t yields a straight line with a R² value of 0.983, and a rate constant k_2_ of 0.006 g/mg.min. The results indicate that the pseudo-second order is more convenient than the pseudo-first order. According to results, adsorption kinetic was determined as the pseudo-second order model owing to its higher R^2^ (correlation coefficients) of **0.983** and a smaller difference between q_e(cal)_ of **30.017** and q_e(exp)_ of **30.432** as compared to the pseudo-first order model **q**_**e (cal.)**_ mg/g **of 29.387** and q_e(exp.)_ of **30.432**, which suggested that the adsorption system follows pseudo second order model.

**Fig. 15:**
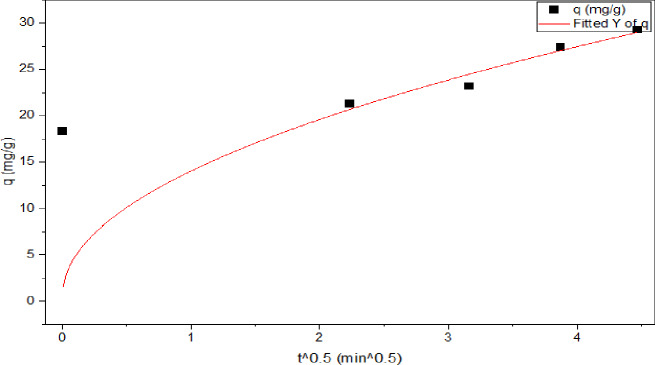
Intra particle diffusion model.

For intra-particle model of weber and Morris (IPD) was used to clarify the adsorption mechanism. Intra-particle diffusion contestant **K**_**id**_ mg/g.min^1/2^ equal **13.141 and** R^2^ (correlation coefficients) of 0.917 with C intercept of 0.636. According to this latter, a plot of q_t_ versus t^1/2^ should be linear if intra-particle diffusion is involved in the adsorption process, and if this line passes through the origin the intra-particle diffusion is the rate controlling step. As can be seen from Fig. 15, C Don’t equal zero and it does not pass through the origin, which indicates that the boundary layer diffusion was a rate control-ling step^71^.

The pseudo-second order model is based on the assumption that the rate limiting step can be physisorption involving valence forces through sharing or exchanging electrons between ad-sorbates and adsorbent. According to the pseudo-second order model, the adsorption rate of 2,4-DNP on MAS/CS NPs was primarily affected by the availability of adsorption sites (functional groups) on the sorbent.

According to the results in Table 2, it can be seen that the pseudo-second order model seems to be suitable for modeling the adsorption of MAS/CNPs due to high R^2^ value and low X^2^ value^72–74^.

***Elovich Kinetic Model***.

The Elovich model proposes that chemisorptions, or chemical reactions, are most likely the mechanism that determines the rate of adsorption. This model Supplementary Fig. 1, may be successfully employed in liquid solutions and the linear form of the Elovich equation Eq. (8).

Is:

8 $$\:{{q}}_{{e}}=\left(\frac{1}{{\upbeta\:}}\right)\mathbf{ln}\:{\upalpha\:}{\upbeta\:}+\left(\frac{1}{{\upbeta\:}}\right)\mathbf{ln}\:\mathbf{t}\:$$

Where, α [mg/g] is the initial sorption rate and β [g/mg] is the desorption constant. The values of α and β can be calculated from the slope and intercept of the plot of q_t_ versus lnt. As supplementary Fig. 1, which explains the relation between q and Ln t as follows. β = 0.130 with intercept of 2.169 with R^2^ = 0.845.

The pseudo second order model’s R² values are closer to unity compared to the pseudo first order and Elovich models. Thus, the pseudo-second order model governs the adsorption of total adsorbates onto an adsorbent. Furthermore, values of q_e_ (cal) predicted using the pseudo-second order model were in good agreement with actual values of q_e_ (exp), indicating that the pseudo-first order and Elovich models are insufficient to represent the kinetics of adsorption^75,76^.

**Table 2:** Parameters characterizing the adsorption kinetics.

Pseudo first order	q_e(exp.)_ = 30.432 mg/g qe(cal.) = 29.387 mg/g K_1_= 0.173 1/min *R*^2 =^ 0.960 X^2^ = 4.350
Pseudo-second order	**q**_**e(exp.)**_: **30.432** mg/g **q**_**e(cal.)**_ **= 30.017** mg/g **K**_**2 =**_ **0.006** g/mg.min **R** ^**2=**^ **0.983** **X**^**2**^ **= 2.352**
Intra-particle diffusion	**C = 0.636** **K**_**id**_**=13.141** mg/g.min^1/2^ **R** ^**2=**^ **0.917** **X**^**2**^ **= 1.125**

### Adsorption isotherm

Many adsorption isotherm models, including the most well-known as Langmuir, Freundlich, DKR and Temkin models, have been employed to explain experimental data.

**Fig. 16:**
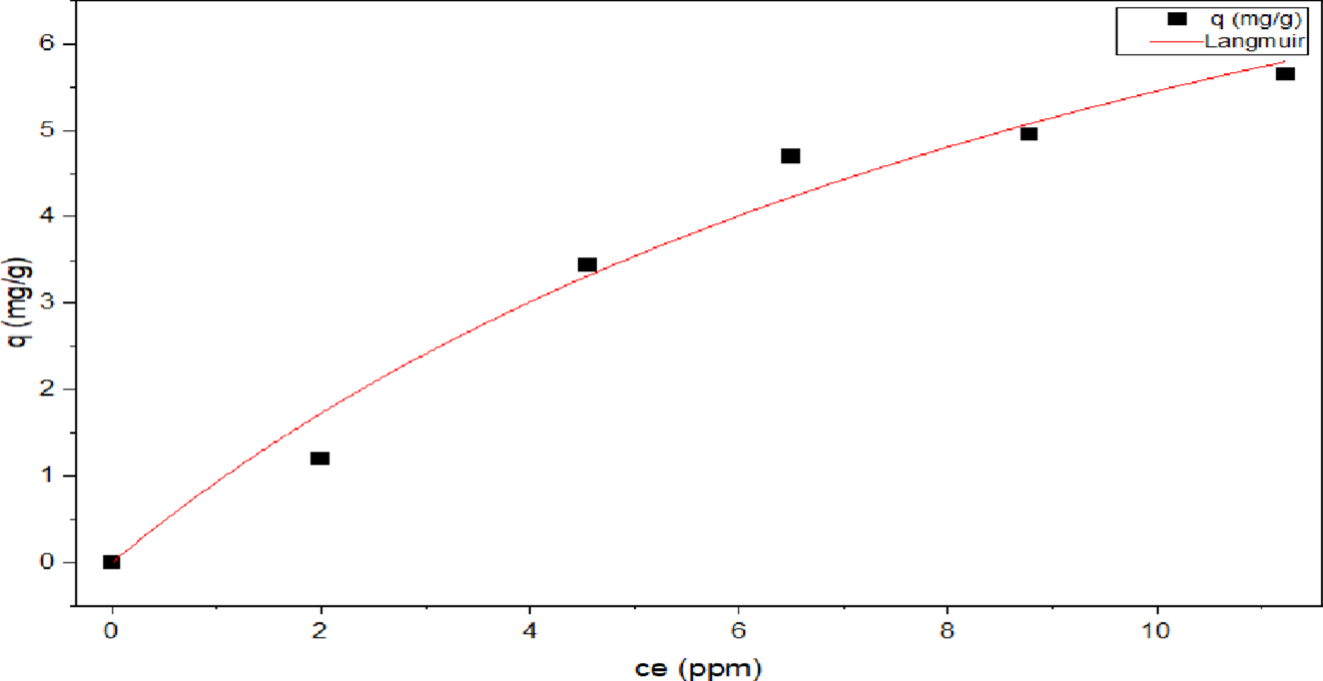
Langmuir adsorption isotherm.

According to the Langmuir isotherm, Fig. 16, a single adsorbate monolayer covering the adsorbent surface has the highest adsorption capacity^77–79^. Adsorbate-adsorbate interactions are not included in this theory since all adsorbate molecules have an equal chance of sticking to the adsorbent surface because of surface homogeneity (Eq. 9) represented the Langmuir isotherm based on these assumptions^80,81,98^.

9 $$\:{{q}}_{\mathbf{e}}={{q}}_{\mathbf{m}\mathbf{a}\mathbf{x}}{*}\frac{{b}{{c}}_{{e}\:}}{1+{b}{{c}}_{{e}\:}}\:\:$$

The linear form (Eq. 10) is the following:

10 $$\:\frac{{{c}}_{{e}}}{{{q}}_{{e}}}=\frac{1}{{{q}}_{\mathbf{m}\mathbf{a}\mathbf{x}}{*}{b}}+\left(\frac{1}{{{q}}_{\mathbf{m}\mathbf{a}\mathbf{x}}}\right){{c}}_{{e}\:}$$

Where q_m_ is the maximal adsorption capacity (the number of dyes required to form one monolayer in mg/g) and K_L_ is the Langmuir constant in (L/mg). (Eq. 11) states that the separation factor of the Langmuir module, or R_L_ for short, is a dimensionless factor.

11 $$\:{{R}}_{{L}}=\frac{1}{1+{b}{C}_{\circ}}$$

Where C_0_ is the highest dye concentration, a favourable reaction occurs if 0 < R_L_ <1, and an unfavourable adsorption reaction occurs if R_L_ > 1. A linear relationship is indicated if R_L_ = 1, and an irreversible adsorption response is indicated if R_L_ = 0^77^.

According the Langmuir model, the Langmuir separation factor (R_L_) indicates if the adsorption process is irreversible (R_L_ = 0), favorable (0 ˂R_L_˃1), linear (R_L_ = 1) or unfavorable (R_L_> 1). For the value range of concentrations studied (2.5–25 ppm), the R_L_ values were 0.144–0.559 that confirms the favorability of 2,4-DNP adsorption onto MAS/CS-NPs with maximum adsorption capacity q_m_ = 11.841 mg/g.

**Fig. 17:**
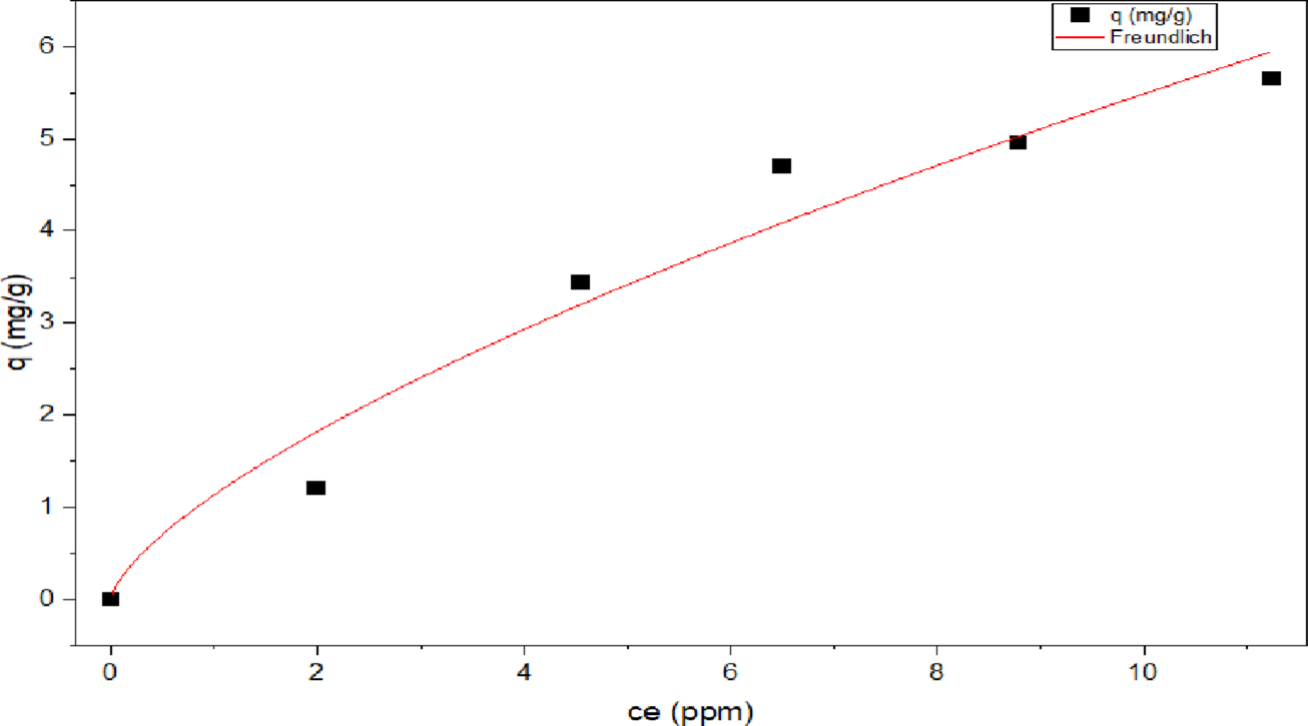
Freundlich adsorption isotherm.

In reference to the Freundlich isotherm module, Fig. 17, it was proposed that adsorbate molecules will interact with each other in addition to the adsorbent surface. The heat of adsorption is not dispersed uniformly due to the heterogeneous nature of the adsorbent’s surface^82–84^ (Eq. 12) is a representation of the Freundlich isotherm equation:^85^.

12 $$\:{{q}}_{{e}}={{K}}_{{f}}{{C}}_{{e}}^{1/{n}}$$

To linearize, use the following constants and logarithms:

13 $$\:{log}\:{{q}}_{{e}}={l}{o}{g}\:{{k}}_{{f}}+\frac{1}{{n}}{log}\:{{C}}_{{e}}$$

Where C_e_ is the equilibrium dye concentration (ppm), K_F_ is the Freundlich constant (1/g), n is the Freundlich constant, and qe is the equilibrium adsorption capacity (mg/g). K_F_ is associated with the extent of adsorption, whereas 1/n is associated with the intensity of adsorption, varies with the heterogeneity of the material, and represents the interaction between the adsorbed species. The experimentally recorded values of n are frequently greater than unity, showing the presence of repulsive forces between the adsorbed molecules; if n is near zero, the system is considered more varied. The values of 1/n and K_F_ are determined by the slope and intercept of the ln q_e_ vs. ln C_e_ plot.

Further, according to the Freundlich model, the exponent n determines the adsorption effectiveness. According to the results, the values of n were higher than 1, the value of *n* = 1.470 suggesting that 2,4-DNP was favorably adsorbed by the MAS/CS-NPs in the concentration range studied.

**Fig. 18:**
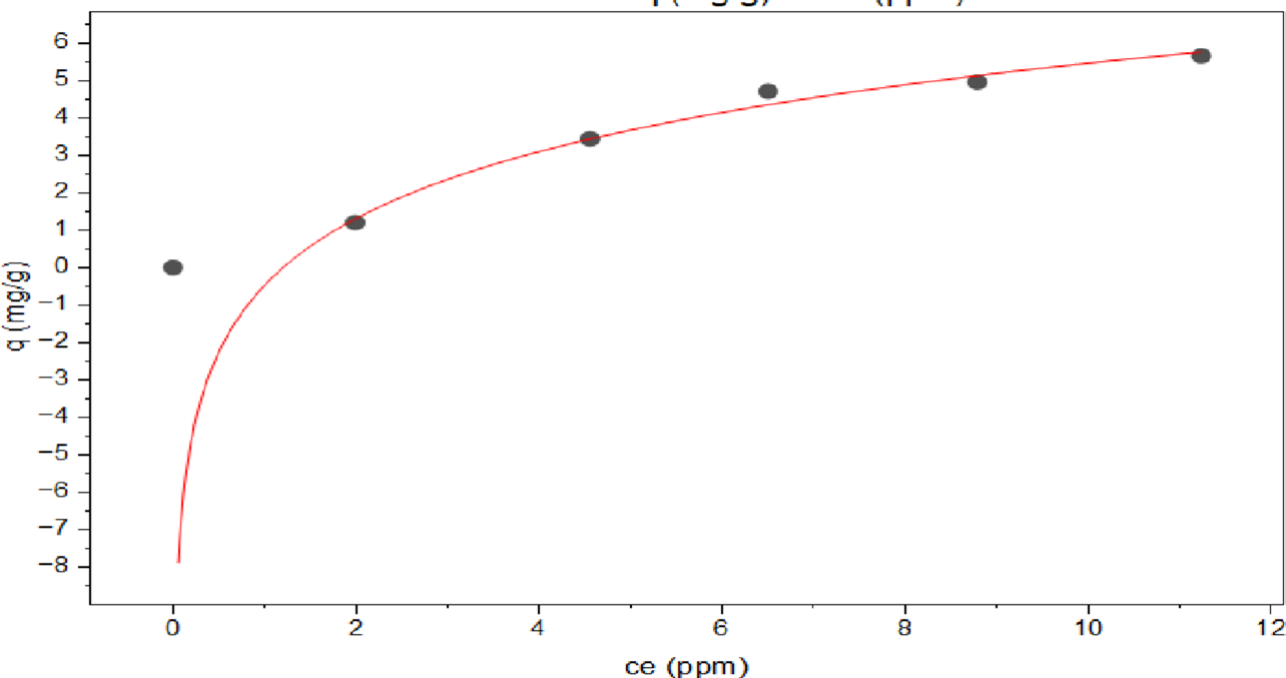
Temkin adsorption isotherm.

In reference to the Temkin isotherm, Fig. 18, two presumptions underlie this isotherm. Firstly, it assumes that the adsorption heat of the whole layer of molecules drops linearly as the coverage rises, which is due to the interactions between the absorbent and adsorbate^86,87^. Moreover, a consistent distribution of binding energies that approaches the maximal binding energy characterizes the adsorption. The Temkin isotherm is widely used for non-uniform sorption heat distribution (Eq. 14)^88^.

14 $$\:{{q}}_{{e}}=\frac{{R}{T}}{{b}}{ln}\:{A}+\frac{{R}{T}}{{b}}{ln}\:{{C}}_{{e}}\:,\:{{q}}_{{e}}={B}{ln}\:{A}\:{C}{e}$$

The equilibrium binding constant, A (L/mol), reflects the maximum binding energy. B is the adsorption’s heat constant where B_T_ = RT/*b* constant associated with the heat of sorption (J/mol) calculated from the Temkin plot (Q_e_ against ln C_e_ ); R = universal gas constant (8.314 J/mol.K), T = temperature (298 K). The Temkin isotherm was obtained straight line. Temkin isotherm constant (*b*) can be obtained from the slope, B_T_ = RT/*b*, and the Temkin isotherm equilibrium binding constant (A) can be obtained from the intercept, (RT/*b*) lnA.

The Temkin constant, B, is related to the heat of adsorption, which indicates if the adsorption reaction is endothermic (B < 1 J mol^− 1^) or exothermic (B > 1 Jmol^− 1^). The B values were 2.577 Jmol^− 1^, that indicating the exothermic nature of 2,4-DNP adsorption onto MAS/CS-NPs.

**Fig. 19:**
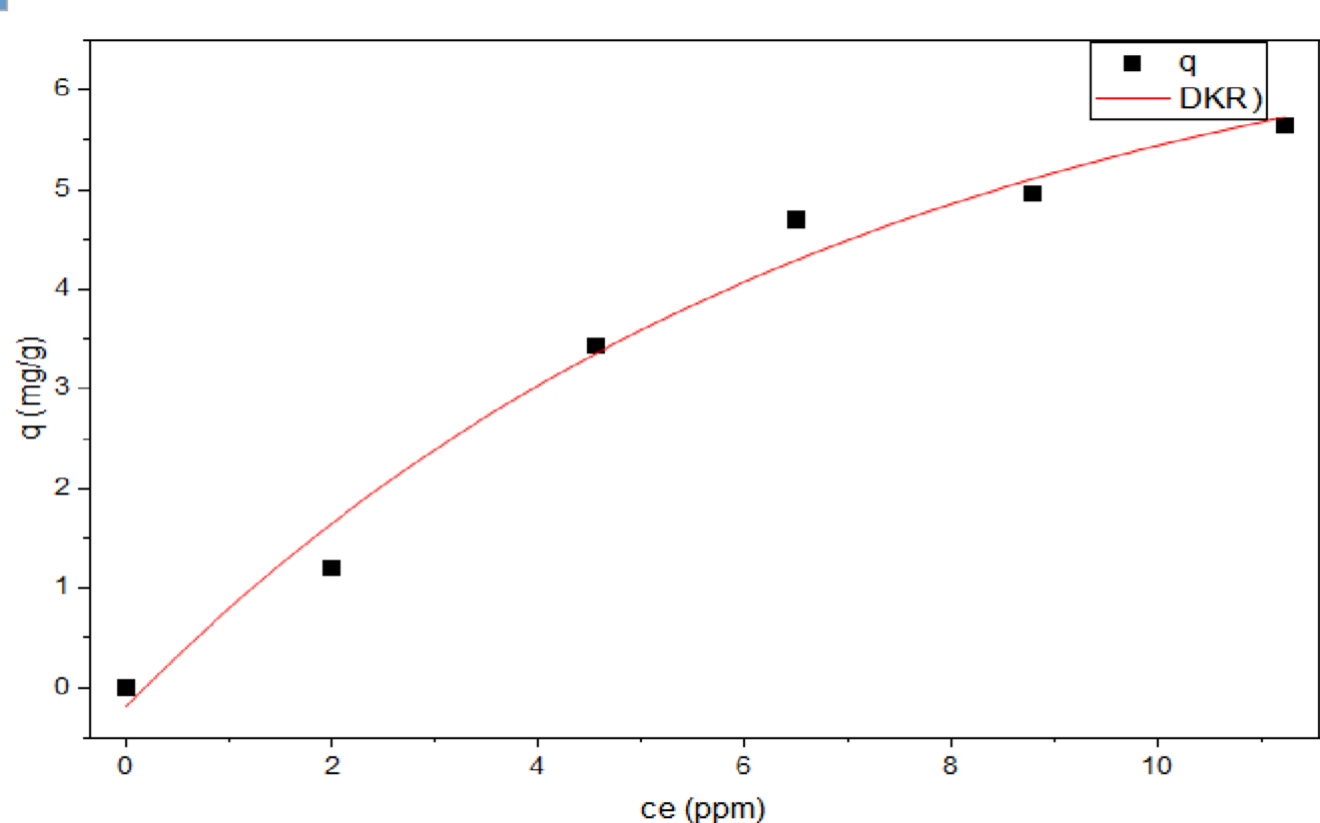
DRK adsorption isotherm using MAS/CSNPs nanopowder.

In reference to the Dubinin-Radushkevich (DKR) isotherm, Fig. 19, is an empirical model and more comprehensive than the Langmuir isotherm as it does not make assumptions about a uniform surface or consistent adsorption potential. It describes the adsorption of MAS/CSNPs on macroporous substances, explicitly focusing on pore-filling. It distinguishes between physical and chemical adsorption to release a molecule from its location in the sorption space to an infinite distance^89–91^. The isotherm has the following linear form and is used to calculate the apparent energy of MAS/CSNPs adsorption onto adsorbent (Eqs. 15 and 16)^92^.

15 $$\:{ln}\:{{q}}_{{e}}={ln}\:{{q}}_{{max}}-{\beta\:}{{\varepsilon\:}}^{2}\:,\:{q}{e}={{q}}_{{max}}\:{{e}}^{-{\beta\:}\left[{R}{T}{l}{n}(1+1/{c}{e})\right]}$$

16 $$\:{\varepsilon\:}={R}{T}{ln}\left(1+\frac{1}{{{c}}_{{e}}}\right)$$

Where $\:{\epsilon\:}$ denotes the Polanyi potential, q_max_ denotes the sorption capacity (mg/g). *β* estimates the average free energy (*E*) (kJ/mol) of sorption for each sorbate molecule moved from infinity in the solution to the surface of the solid as shown in the following (Eq. 17).

17 $$\:{E}=\frac{1}{\sqrt{2{\beta\:}}}$$

The type of adsorption process is mostly dependent on the value of the mean free energy (*E*), if 8 < *E* < 16 kJ/mol the adsorption process is ion exchange, if E < 8 kJ/mol, the adsorption process is physical interaction, if E > 16 kJ/mol the adsorption process is chemical interaction.

From data was applied for DRK model, and straight lines were obtained when the DKR isotherm was plotted lnq_e_ against $\:{\epsilon\:}$^2^ with E = 0.351 kJ/mol, qs = 5.650 mg/g and R^2^ = 0.914. The outcome of the mean adsorption energy derived from the Dubinin-Radushkevich isotherm reveals that the adsorption mechanism is primarily physisorption rather than chemisorption. This is indicated by an *E* value below 8 kJ/mol, which signifies that physisorption controls.

As presented in Table 3; Figs. 16, 17, 18 and 19, **t**he values of the parameters for the four isotherms and the accompanying correlation coefficients where a comparison is also made between the four isotherms and showed that the isotherm is fitted with Tamkin model which have the higher R^2^ value = 0.988 with lowest X^2^ of 0.058^93,94^.

**Table 3:** The isotherm parameters for the adsorption.

Adsorption isotherm	Values	Adsorption iIsotherm	Values
Langmuir	q_max_=11.841 mg/g K_L_ =0.085 L/mg R^2^ = 0.961 X^2^ = 0.182	**DKR**	E = 0.351 kJ/mol q_max_=5.650 mg/g R^2^ = 0.914 X^2^ = 0.437
Freundlich	*n* = 1.470 k_f_=1.135 (mg/g) (L/g)1/n R^2^ = 0.902 X^2^ = 0.301	**Temkin**	B = 2.577 J/mol A = 0.829 L/mg R^2^ = 0.988 X^2^ = 0.058

### Thermodynamic study

The spontaneity of an absorption process is evaluated using thermodynamic characteristics. The following equations were used to calculate the changes in the absorption processes free energy (ΔG°), enthalpy (ΔH°), and entropy (ΔS°) utilizing the subsequent Eqs. (18–20):

18 $$\:{{K}}_{{d}}=\frac{{{q}}_{{e}}}{{{C}}_{{e}}},$$

19 $$\:\varDelta\:{{G}}^{{o}}=-{R}{T}{l}{n}{{K}}_{{d}},$$

20 $$\:{n}{{K}}_{{d}}=\frac{-(\varDelta\:{{H}}^{{o}})}{{R}{T}}+\frac{\varDelta\:{{S}}^{{o}}}{{R}},$$

C_e_ stands for the equilibrium concentration of the remaining dye in the solution, whereas qe is the equilibrium concentration of the adsorbed dye on the adsorbent. A Van’t Hoff plot of the ln kd shows a linear relationship vs. the temperature reciprocal (1/T)) Supplementary Fig. 2. The slope and intercept of the line can be used to get the values of ΔH° and ΔS°. Their -ve values indicate the exothermic nature of the absorption onto the composite and reduce the randomness due to adsorption as ΔH° value of − 39.479 kJ/mol, and ΔS° value of − 102.653 kJ/mol.K. At various temperatures (298, 308, and 313 K), the ΔG° values were – 7.203, − 6.931, and – 5.514 kJ/mol, respectively (as supplementary Table 1). These values suggest that DNP absorption occurs spontaneously^95,96,97^.

### The adsorption mechanism

A multi-mechanism technique for the removal of 2,4-DNP by the MAS/CS-NPs composite is developed based on the thorough characterization and adsorption data. The collaboration of MAS spinel and chitosan nanoparticles enables the following distinct interactions:

1. **Electrostatic Attraction** (The Principal Mechanism): This is recognized as the predominant mechanism, substantiated by the pH analysis (Sect. 3.4.1, Fig. 6). Under acidic circumstances (pH = 4, the optimal pH), the surface of the MAS/CS-NPs composite becomes protonated and acquires a positive charge. Simultaneously, the 2,4-DNP molecule experiences deprotonation of its phenolic -OH group, resulting in the formation of a phenoxide anion. The strong electrostatic interaction between the positively charged adsorbent surface (e.g., -NH₃⁺ sites on chitosan) and the anionic 2,4-DNP molecule is the principal factor for the elevated adsorption capacity.
2. **Hydrogen Bonding**: FTIR study (Sect. 3.3, Fig. 3) offers direct evidence for this mechanism. The alterations in the characteristic bands upon adsorption validate contact.
  The broad bands at 3398 cm⁻¹ and 3762 cm⁻¹, corresponding to N-H and O-H stretching, exhibited shifts and alterations in form, signifying their participation in hydrogen bonding between the composite and 2,4-DNP dye.
  The N-H bending vibration (amide II) at 1557 cm⁻¹ was shifted to 1559 cm⁻¹, indicating that the amino groups of chitosan engage in hydrogen bonding with the adsorbate.
  Consequently, the -OH and -NH₂ groups on the chitosan matrix function as hydrogen donors, whilst the -NO₂ and -O⁻ groups on 2,4-DNP serve as hydrogen acceptors^50,54^.

3. **Pores and Physical Adsorption (Physisorption)**: The structure characteristics validated by BET analysis (Sect. 3.5, Table 1) indicate that the best composite (1:9 ratio) possesses a substantial surface area (82.88 m²/g) and a mesoporous characterizes. This enables the diffusion and physical trapping of 2,4-DNP molecules within the pores. Additionally, the Dubinin-Radushkevitch (D-R) isotherm model produced an adsorption energy (E) of 0.351 kJ/mol (Sect. 3.6, Table 3). This number, being substantially lower than 8 kJ/mol, demonstrates that the entire process is mostly dictated by physisorption (van der Waals forces) rather than chemisorption.
4. EDX analysis (Sect. 3.4, Fig. 5) identified the presence of Ni and Tb dopants inside the composite. The presence of these Lewis acid metal centers (Ni²⁺, Tb³⁺) on the MAS surface and the Lewis basic nitro groups on 2,4-DNP suggests that weak coordinative interactions could serve as a secondary, contributing mechanism. This minor chemisorptive component is consistent with the kinetic model but is not the primary driver of adsorption, as evidenced by the low E value from the D-R isotherm.

The adsorption mechanism commences with the fast diffusion of 2,4-DNP anions towards the composite surface, propelled by electrostatic attraction. Upon contact, the molecules are further stabilized through hydrogen bonding with the functional groups of chitosan and by filling the pores inside the mesoporous structure. The presence of weak physisorption forces is evidenced by the low E value, although possible coordination with metal dopants introduces further complication. The synergistic interplay of processes elucidates the elevated removal efficiency, rapid kinetics, and significant capacity of the MAS/CS-NPs (1:9) composite for 2,4-DNP.

### Elution and regeneration cycles

**Fig. 20:**
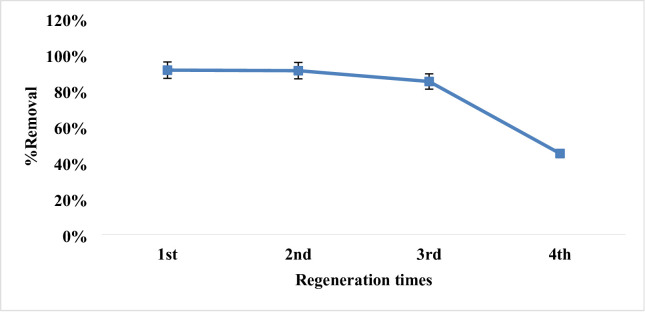
Regeneration cycles of adsorbent.

Regeneration of the adsorbent should be done to lower the cost of the removal process. After each adsorption cycle, when the MAS/chitosan nanopowder was saturated, adsorption phase was performed in order to replenish the biosorbent material. The sorbent was rinsed with water after each stage, followed by 0.01 mol L^− 1^ of NaOH and HCl for the 2,4-DNP dye. In this investigation Fig. 20, adsorption and desorption occurred four times in a row. For 2,4-DNP dye, regeneration efficiency was determined to be 85% up to 3 cycles. These efficiency measures were provided for three standard cycles. These results demonstrated the synthetic MAS/chitosan nanopowders capacity for regeneration^9,10^.

### Comparative studies

Table 4 shows the comparative study about the adsorption removal of 2,4- DNP using different adsorbent dosage, pH, contact time, with their adsorption capacity. As the following data the composite NPS can give high adsorption capacity refer to the adsorbent dose 0.03 g through just 45 min with removal about 91.3% on contrast the other studies need long time about 1 h or large adsorbent dosage.

**Table 4:** Comparison the different parameters of the previous studies of 2, 4-DNP removal.

Adsorbent	Removal (%)	pH	Dosage (g/l)	Time (Min)	Temp. (K)	Isotherm	Kinetics	Ref.
Magnetic Nanoparticles with Polypyrrole (MNPs-PPy)	98.67	6	0.05	75	318	–	–	^60^
Commercial activated carbon	74–71	4	0.2	300	–	Freundlich		^99^
Amine functionalized metal organic framework (NH_2_ -MIL-101(Cr)	98.9	4	0.5	50	–	Langmuir isotherm.	Pseudo second order	^100^
Active carbon	99.0	4	0.15	60	–	Langmuir isotherm	Pseudo-second-order model	^101^
MAS/CSNPs nanopowder	91.3	4	0.03	45	**Room temperature**	Temkin isotherm	Pseudo-second-order model	**This study**

## Conclusion

Shrimp shells were employed to prepare chitosan nanopowder, alongside citric acid, aluminium, and magnesium, to efficiently synthesize a single nanocrystalline magnesium aluminate spinel powder (MgAl_2_O_4_: _0.0011_Tb^3+^: _(0.1)_ Ni^2+^) via the sol-gel auto-combustion technique. XRD analyses were employed to characterize the synthesized nanoparticles. XRD analysis of spinel powders and MAS/CSNPs indicated that the material is nanocrystalline, while TEM analysis demonstrated that the synthesized nanomaterials possess well-developed nanoscale dimensions. These materials were utilized as adsorbents for the removal of 2,4-DNP, and it is evident that 2,4-DNP can be effectively eliminated from wastewater using a MAS to chitosan nanoparticle weight ratio of 1:9. The 2,4-DNP dye was eliminated using MAS/chitosan nanopowders, achieving a removal efficiency of 91.3% with 0.03 g/L of adsorbent at pH 4, room temperature, and a contact duration of 45 min. The examination of isotherm and kinetic models indicated that the experimental data conformed closely to the pseudo-second-order model and Temkin isotherm, to boundary layer diffusion identified as the rate-limiting step.

## Supplementary Information

Below is the link to the electronic supplementary material.

Supplementary Material 1

## Data Availability

All the authors confirm that the data supporting the findings of this study are available within the article.
