# Supplementary material for: Removal of 2,4 di-nitrophenol by using modified spinel aluminate/chitosan nanoparticles composites
Source: Sci Rep. 2025 Dec 3;15:43108. doi: 10.1038/s41598-025-28057-8 (PMC12678815; doi:10.1038/s41598-025-28057-8)
Supplement: Supplementary file 1 — Supplementary Material 1 [file 41598_2025_28057_MOESM1_ESM.docx]

**Removal of 2,4-dinitrophenol by using modified spinel aluminate/ chitosan nanoparticles composites**

**Amany N. Georgy**^1*^**, Mohamed A. Omar** ^1^**,** **Maysa R. Mostafa** ^1^**, Gehad G. Mohamed^1,2^, Omar A. Fouad** ^1^

*^1^ Department of Chemistry, Faculty of Science, Cairo University, Giza 12613, Egypt*

*^2^ Nanoscience Department, Basic and Applied Science Institute, Egypt-Japan University of Science and Technology, New Borg El Arab, Alexandria, 21934, Egypt*

**The corresponding authors**

**Amany N. Georgy:** [**kirasnasry@yahoo.com**](mailto:kirasnasry@yahoo.com)

**Materials**

**Materials and solutions**

At the analytical level, every component was utilized exactly as intended. Magnesium nitrate (Mg(NO_3_)_2_.6H_2_O; Merck), aluminium chloride (AlCl_3_.6H_2_O; 97%), citric acid monohydrate (C_5_H_8_O_7_.H_2_O;98%), hydrochloric acid (HCl; 37%), ethylene glycol (99%), ammonia solution (33%; Sigma–Aldrich Chemical Co.), nickel nitrate (Ni(NO_3_)_2_.6H_2_O), terbium oxide ( Tb_4_O_7_;99%, Sigma–Aldrich Chemical Co.), The source of hydrochloric acid (HCl) and sodium hydroxide (NaOH) were Honeywell, Germany. Shrimp shells from market, Sodium hydroxide (NaOH;99%), and 2,4-DNP(2,4-DNP dye C_5_H_4_N_2_O_5_; 184 g mol^-1^) was bought from Sigma-Aldrich. Ethyl alcohol are from Sigma-Aldrich Chemie GmbH, which is based at Eschenstrasse 5 D-82024 TAUFKIRCHEN. The ammonia solution came from the German company Riedel-deHaen.

**Instruments**

Using a Bruker D8 Discover X-ray diffractometer, the phase composition of the synthesized nanoparticles and sintered samples was ascertained using Ni-filtered Cu K radiation (= 1.5406). N2 has been employed as the adsorptive gas in gas adsorption investigations in order to calculate the BET surface area at 77 K. The materials were evacuated under a high vacuum for four to twelve hours prior to the adsorption test. The Brunauer-Emmett-Teller (BET) hypothesis served as the basis for the computation, and a Nova Touch LX2 analyzer was used for the study. The microstructure and pore size distribution of a  selected samples were examined using a scanning electron microscope (SEM) of cracked surfaces utilizing the Philips XL30 model, an accelerating voltage of 30 kV, magnification up to 400000, and resolution for W [3.5 nm]. Samples were lightly coated in gold prior to testing. Transmission electron microscopy was used to analyze the size and form of the produced nanoparticles (TEM; JEOL JEM-2100, Tokyo, Japan). The quantities of the dyes under study were determined using a spectrophotometric method. Plotting the absorbance versus concentration at a maximum wavelength of 360 nm for 2.4. Dinitrophenol allowed for the creation of calibration curves using a UV-vis spectrophotometer. The final concentration was determined spectrophotometrically in accordance with the dye λmax using the Beer- Lambert equation.

Supplementary Figure 1 Elovich kenetic parameter.

Supplementary figure 2. Thermodunamic factors with vanthoff plot

Supplementary table 1. Thermodynamic parameters of the 2,4 DNP by the composite

| Temperature (K) | ΔG^0^  (KJ/mol) | ΔH^0^  (KJ/mol) | ΔS^0^  (J/mol/K) |
| --- | --- | --- | --- |
| 298 | -7.2031 | -39.479 | -102.653 |
| 308 | -6.9312 |  |  |
| 313 | -5.51487 |  |  |
